# Use of the Italian version of the Pediatric Asthma Quality of Life Questionnaire in the daily practice: results of a prospective study

**DOI:** 10.1186/1471-2431-9-30

**Published:** 2009-05-07

**Authors:** Giampaolo Ricci, Arianna Dondi, Elena Baldi, Barbara Bendandi, Arianna Giannetti, Massimo Masi

**Affiliations:** 1 Department of Pediatrics, University of Bologna, Italy; 2 Medicine and Public Health Department, University of Bologna, Italy

## Abstract

**Background:**

Asthma is a serious global health problem and its prevalence is increasing, especially among children. It represents a significant social and economic burden, and it can severely affect the health-related quality of life (HRQL) of patients. Among the numerous questionnaires aiming at evaluating asthma HRQL in children, the Pediatric Asthma Quality of Life Questionnaire (PAQLQ) has proved to have good measurement properties.

The present study was aimed at investigating the possible role of the Italian, self-administered version of the PAQLQ in the routine clinical evaluation of children affected by bronchial asthma.

**Methods:**

52 Italian children and adolescents (40 males and 12 females), aged 6 to 17 years, affected by allergic asthma, were enrolled. Each patient was evaluated twice, and at each visit asthma control and severity were assessed, spirometry was performed and the patients completed the self-administered version of the PAQLQ.

**Results:**

The questionnaire was well-accepted and understood by the children. Children showed an overall good quality of life, with mild impairment in the activity and emotional function domains. The PAQLQ showed an overall good correlation with the clinical and functional indexes that are normally evaluated in follow-up visits of asthmatic patients. The PAQLQ appeared to be strongly related to asthma control, both at the first (p < 0.01) and second (p < 0.001) time of the study. The PAQLQ was also seen to decrease with increasing asthma severity. The results suggest a better compliance of the children towards completion of the questionnaire at t_1_. Finally, the PAQLQ does not appear to discriminate HRQL in patients with good lung function.

**Conclusion:**

The Italian version of the PAQLQ is a quick-to-administer aid to clinical activity and can add valuable information to symptom reports, objective measurements and clinical assessment of asthma control and severity in daily clinical practice. Re-administration at each follow-up visit allows HRQL to be monitored over time.

## Background

The role of the evaluation of patients' health-related quality of life (HRQL) has gained importance since 1948, when the World Health Organization defined health as being not only the absence of disease and infirmity, but also the presence of physical, mental and social well-being [1,2]. HRQL measurement is therefore a multidimensional assessment and it usually includes physical functioning and somatic sensation, as well as social and emotional functioning and well-being [3,4]. The most famous definition of quality of life is the one by Schipper et al. [5]: "Quality of life in clinical medicine represents the functional effect of an illness and its consequent therapy upon a patient, as perceived by the patient". The interest in considering HRQL as an important tool for a thorough comprehension of the child's health is increasing, as demonstrated by the recent publication of a survey on almost 70.000 children to evaluate HRQL in the pediatric population of the United States [6].

Asthma is the most common chronic disease in childhood in nearly all industrialized countries [7], seriously interfering with patients' HRQL [8] and imposing a huge burden on the patients, their families and society [9,10]. Pediatric asthma accounts for a large proportion of childhood hospitalizations, healthcare visits, absenteeism from day care/school and missed work days by parents [11]. The more severe the asthma, the worse the patient's HRQL seems to be [12]. However, even mild asthmatics suffer on account of their condition: the level of anxiety has been recognised as comparable in children with mild and severe asthma [13,14].

HRQL assessment in children and adolescents with asthma is an important tool to use if we wish to reduce the current gap in understanding that exists between health professionals' knowledge of the physiological correlates of asthma and the individual burden of experiencing asthma [3]. Parents do not perceive their children's HRQL accurately, hence it is necessary to obtain the information directly from the child [15].

Several instruments have been developed to evaluate asthma-related quality of life. The Pediatric Asthma Quality of Life Questionnaire (PAQLQ) [16] is a 23-item measure for the age range 7–17 and it investigates three domains: the symptoms, the limitations in activity and the emotional function. Its measurement properties were evaluated in a study which involved 52 children with a wide range of asthma severity. It was recognized to have a good construct validity, responsiveness to change over time and test-retest reliability [3]. The PAQLQ has been translated into several languages: its Italian version was proposed in 1999 [12] and the cross-cultural adaptation was made according to the proposed guidelines [17].

The aim of the present study was to evaluate the correlation between the Italian, self-administered version of the PAQLQ and the main clinical and instrumental indexes that are normally included in the outpatients' routine visits, and to investigate the possible role of the questionnaire during routine clinical practice visits of asthmatic children.

## Methods

### Study plan

This prospective study included 52 consecutive children (median age 11 years; range 6–17; 40 males and 12 females), affected by allergic asthma and referred to the Allergology Outpatient Clinic of the Pediatric Department of our hospital. Out of the total number, 23 (44%) were found to be affected by seasonal type asthma, related to symptoms appearance during spring time, while the remaining 29 (56%) had perennial asthma, unrelated to specific periods. Each child had a comprehensive evaluation at his/her first visit which included: a) anamnestic recordings and full physical inspection; b) spirometric assessment of lung function; c) HRQL assessment, by means of the PAQLQ; d) asthma severity assessment; e) asthma control assessment. A follow-up visit was decided depending on the child's type of asthma: children with perennial asthma had a second evaluation after 6 months if their health status was normal, and after 1 month if they were not controlled or if their spirometry was altered; children with seasonal asthma attacks, who had been included before springtime, were examined again during the spring.

### Asthma control evaluation

For the assessment of asthma control, long-term treatment objectives identified by the GINA working group [18] were adopted. The definition of asthma control was derived from these goals, as suggested by Bateman [19] and by the Joint Task Force on Practice Parameters [20]. A child was considered either under control or not under control in the two weeks before the visit according to the following parameters: no night-time or early morning awakening due to asthma; no emergency hospital visits; no exacerbations; no treatment-related adverse effects causing a change in asthma therapy; no more than two days with symptoms (cough, wheezing, chest tightness, dyspnea); use of β_2_agonist reliever medications for no more than two days.

### Spirometry

Lung function was assessed by means of a spirometric analysis (Multispiro SA/100 Spirometer, Medical Equipment Designs, Laguna Hills, CA). At spirometry, the following parameters were evaluated: forced vital capacity (FVC); forced expiratory volume at the first second (FEV_1_); percent ratio FEV_1_/FVC; forced expiratory flow between 25% and 75% of vital capacity (FEF_25–75_).

### Asthma severity

Asthma severity was assessed according to the GINA guidelines (Updated 2005) [21], thus divided into four levels: intermittent, mild persistent, moderate persistent and severe persistent.

### Quality of life

HRQL was assessed by means of the Italian, self-administered version of the PAQLQ [12], which includes 23 questions, further grouped into 3 domains:

1. *symptoms*: questions 1, 3, 5, 7, 9, 11, 13, 15, 17, 20;

2. *activity limitations*: questions 16, 19, 21, 22, 23;

3. *emotional function*: questions 2, 4, 6, 8, 10, 12, 14, 18.

After receiving exhaustive explanations on questions and answering modalities, the children themselves filled out the questionnaire during both visits, being blinded to replies given at the first visit. For each child, the overall score was calculated as the mean of scores obtained for each single question. The scores ranged from 1 to 7, 1 being the minimum and 7 the maximum. The same approach was adopted for subgroups of questions. Differences in the PAQLQ score ≥ 0.5 were considered as significant.

### Informed consent

This study was only observational and did not interfere with the clinical management of the patients, so it was not submitted to the ethical committee for approval. However, both the parents and the patients were informed that the questionnaire was proposed in an experimentational manner; they were given the questionnaire only after obtaining an informed consent.

### Statistical analysis

The Kruskall Wallis test was used to compare the scores of the PAQLQ in five subgroups of patients, split into quintiles (the first group had FEV_1 _lower than the 20^th ^percentile, the second between the 20^th ^and 40^th ^percentile, the third between the 40^th ^and the 60^th ^percentile, the fourth between the 60^th ^and 80^th ^percentile and the fifth higher than the 80^th ^percentile). This approach is consistent with the small population sample (n = 52) and its non-gaussian value distribution. Mann-Whitney test was used to evaluate possible differences between non-paired groups and Wilcoxon test for paired data at t_0 _and at t_1_. Statistical analysis was carried out by means of SPSS 16 for Windows and Study Size Trial 1.08. Results were deemed statistically significant for a p ≤ 0.05.

## Results

### Population characteristics, asthma severity and control, PAQLQ percentiles

The main clinical characteristics of our population are shown in Table 1.

**Table 1:** Characteristics of the 52 children, median age 11 years (range 6–17). ICS: inhaled corticosteroids. Other medications: leukotriene modifiers, chromones, antihistamines.

**Sex**	*Males*	n	40	77%
	*Females*	n	12	23%
**seasonality of asthma**	*Perennial*	n	29	56%
	*spring-time*	n	23	44%
**controller medications**	*ICS*	n	42	81%
	*other medications*	n	24	46%
	*ICS or other medications*	n	46	89%
**exercise-induced asthma**		n	27	52%
**Comorbidities**	*Rhinoconjunctivitis*	n	34	65%
	*present Atopic Eczema*	n	9	17%
	*past Atopic Eczema*	n	20	39%

Table 2 summarizes the main parameters considered in the study.

**Table 2:** Summary of data in the two steps of the study.

	Data at t_0_			Data at t_1_		
	
Clinical evaluations	N (%)			N (%)		
Asthma severity						
1	29 (55,8%)			21 (40,4%)		
2	15 (28,8%)			18 (34,6%)		
3	6 (11,5%)			10 (19,2%)		
4	2 (3,8%)			3 (5,8%)		
Under-control asthma	39 (75%)			32 (61,5%)		

Quantitative measures	Mean (standard deviation)	50^th ^P	25^th ^P–75^th ^P	Mean (Standard deviation)	50^th ^P	25^th ^P–75^th ^P

FVC	105,66 (15,36)	106,60	97,85–116,15	106,21 (12,91)	107,15	99,15–113,60
FEV_1_	100,10 (18,47)	101,90	94,00–112,45	100,95 (17,28)	102,30	90,55–113,15
FEV_1_/FVC	94,10 (12,16)	97,55	88,40–102,75	94,11 (11,37)	96,35	88,60–102,55
FEF_25–75_	94,98 (32,29)	98,85	71,90–120,45	94,64 (34,58)	97,40	68,50–125,10
PAQLQ total score	6,09 (1,04)	6,48	5,85–6,85	6,24 (0,88)	6,41	5,85–6,91
PAQLQ-symptoms	6,79 (1,33)	7,26	5,98–7,81	6,98 (1,12)	7,34	6,46–7,84
PAQLQ-activity limitation	4,77 (1,02)	4,88	4,16–5,68	4,87 (0,94)	5,08	4,08–5,88
PAQLQ-emotional function	5,75 (0,92)	6,23	5,86–6,23	5,90 (0,78)	6,23	5,86–6,23

Asthma severity 1 = Intermittent, 2 = Mild persistent, 3 = Moderate persistent, 4 = Severe persistent.

### Understanding of the PAQLQ

Children had an overall good understanding of the PAQLQ. The main comprehension problems arose concerning the concept of "last week", which was not always clear for the youngest patients (ages 6, 7 or 8 years). For the younger patients, a longer explanation and some kind of assistance by a doctor or nurse was needed, but no help was given by the parents in the replies. In reality the study group included only one child aged 6 years (already going to school) and three children aged 7. Despite the fact that, after exhaustive explanation, they all expressed a good understanding, it could be argued that these factors may cause some biases in the completion of the self-administered version of the questionnaire.

### Evolution of the PAQLQ score and changes in the other parameters between the two visits

Neither Wilcoxon signed rank test nor Student paired test were s.s. for any difference between time 0 and time 1.

No s.s. difference in asthma control but a significant difference in asthma severity evaluation (p < 0.01) was identified at Mc Nemar chi square owing to the worsening of 13 patients: 8 passed from class 1 to class 2 (7 patients) and one of them passed to class 3; 4 patients passed from class 2 to class 3 and 1 patient passed from class 3 to class 4. All the other patients were unvaried.

Regarding the medications used by our population, 15 patients (29%) needed to use salbutamol for asthma attacks between the two visits; most of the patients (80%) also needed steroid courses, either as continuous therapy or as needed.

### Differences in HRQL between t0 and t1 and correlation with asthma control and asthma severity

No significant improvement or worsening in HRQL between the two examinations was identified in our population with the Wilcoxon test (19 patients had a higher score, 29 a lower one and 4 had the same result); spirometric indices did not change either. On the other hand, some modifications between the first and the second visit were identified in asthma severity and control: at the second visit the percentage of moderate and severe persistent asthma is higher (p < 0.02) and a higher number of children are not under control (but this difference is not s.s.).

**Figure 1:**
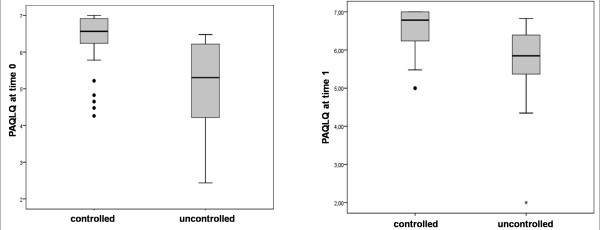
**Differences in HRQL between controlled and uncontrolled patients at the first visit (p < 0.01) and at the second visit (p < 0.001)**.

Results of the Mann-Whitney U test showed a significant difference between asthma control and the PAQLQ score both at the first (p < 0.01) and at the second visit (p < 0.001): children with a good control of symptoms have a better HRQL, as plotted in Figure 1. The same test showed that patients affected by seasonal-type asthma at the first visit had a better HRQL than those affected by non-seasonal-type asthma (p ≤ 0.05); this difference was no longer present at the second visit. At the first visit, patients with spring-related symptoms had a better control than children with non-seasonal-type asthma (p ≤ 0.01), whereas data from the second visit do not show this difference.

**Figure 2:**
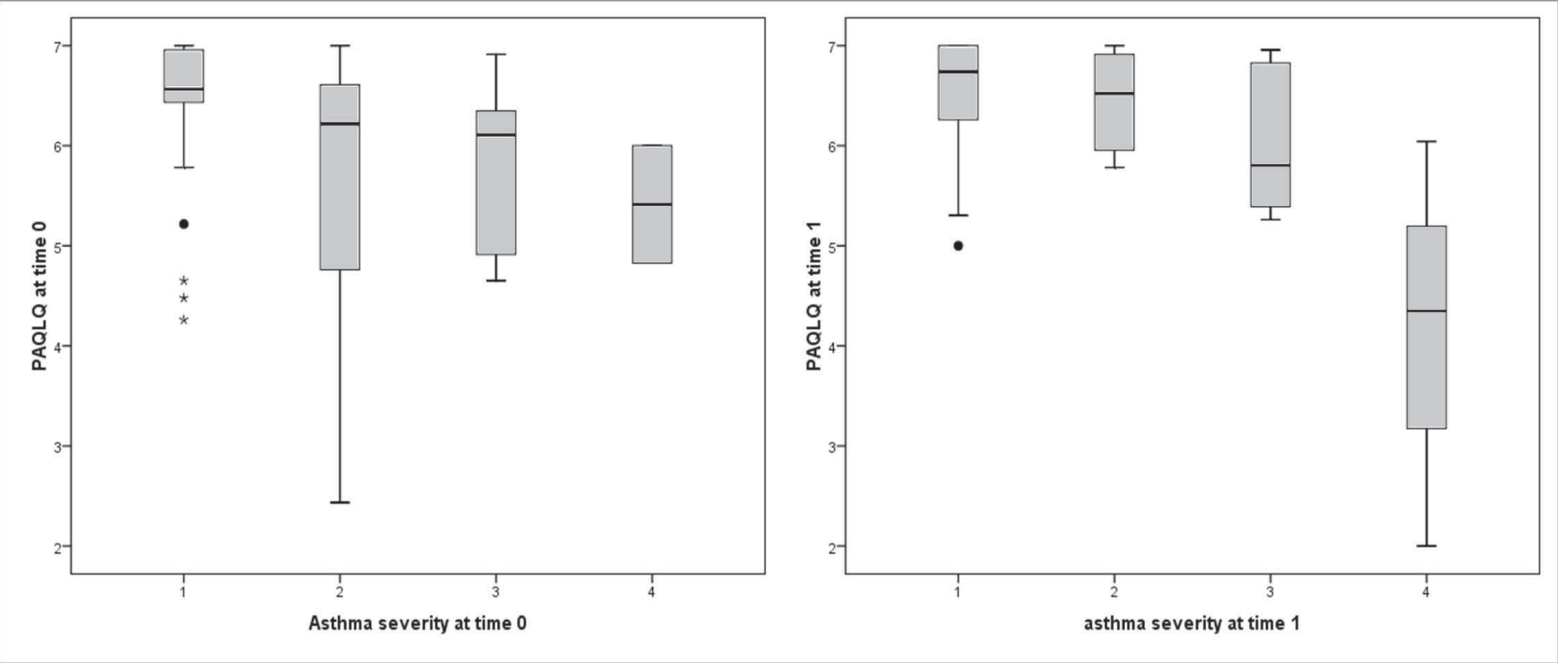
**Decrease in the PAQLQ score in patients with increasing asthma severity at time 0 and at time 1 (p < 0,04 at time 0 and p < 0,03 at time 1)**.

Kruskall Wallis test was s.s. (p < 0.04) for a decrease in the PAQLQ score from the first to the fourth class of asthma severity, both at time 0 and at time 1 (Figure 2). This means that HRQL is worse when the disease is more severe, as could be expected.

**Figure 3:**
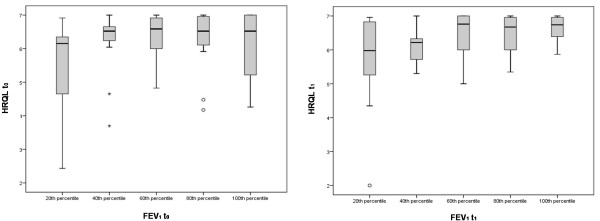
**Median HRQL score trend in function of FEV_1 _values at t_0 _(on the left) and at t_1 _(on the right)**. Boxes are delimited by the 25^th ^percentile (at the bottom) and 75^th ^percentile (top) of HRQL.

To analyze better the relationship between the PAQLQ and lung function, the PAQLQ score was evaluated in 5 subgroups of patients who were divided according to their FEV_1 _(Figure 3). The graphs suggest a better compliance of the children towards completion of the questionnaire at t_1_. Moreover, it seems that the PAQLQ does not discriminate HRQL in patients with a good lung function: figure 3 shows a plateau of the results for patients' FEV_1 _over the 60^th ^percentile.

## Discussion

Our results confirm the link between the Italian version of the PAQLQ and the main clinical and functional parameters which are normally evaluated during follow-up visits of asthmatic school-aged children. Several translated versions of the questionnaire exist in other languages, and they are all consistent with our results.

A study conducted on 52 Canadian children aged 7 to 17 demonstrated that the PAQLQ has an excellent responsiveness and a high level of reliability [16]. The questionnaire showed good levels of both longitudinal and cross-sectional correlations with the conventional asthma indices and with general quality of life. However, no correlations were found between the questionnaire's score and FEV_1 _percent of predicted.

A study that examined the validity of the Swedish version of the PAQLQ was published in the year 2000 [22]. Sixty-one 7-to-9-year-old children were enrolled. Their symptoms score, percentage of expected peak flow rate and physician's grading of asthma severity correlated significantly with the PAQLQ scores. The instrument was found to be easy to administer, well accepted by the children and had an acceptable internal consistency.

Similar results were obtained in the validation of the Spanish cross-cultural adaptation of the PAQLQ [23], which involved 99 children. Correlations between the scores of the PAQLQ, of an asthma control instrument, of a general health questionnaire and lung function indexes were moderate. The Spanish version of the PAQLQ, compared to the original, showed a similar internal consistency, reliability, validity and sensitivity to clinical changes.

In 2005 a study was published which validated the Dutch version of the PAQLQ [24]. 238 children aged 6 to 18 years were evaluated. Correlations between the questionnaire's score and symptom diary scores indicated adequate psychometric properties, excellent responsiveness and supported the longitudinal and cross-sectional construct validity. No lung function parameters were taken into account.

Our results are consistent with those of the above studies: the PAQLQ correlates with the assessment of asthma control given by the clinician. These results indicate that the Italian version of the PAQLQ is a valid instrument that can be used in daily practice when asthmatic children are evaluated. However, the PAQLQ seems to be unable to discriminate HRQL in children with good lung function, suggesting that a more sensitive instrument might be needed in these cases.

The fact that HRQL is worse in children with lower asthma control may seem obvious. However, administration of the questionnaire allowed us to note that the main domains which are impaired in asthmatic children are the *symptoms *and the *activity limitations*. Children and adolescents are often worried about asthma attacks, and cough and chest tightness might be a cause of concern for them. They also seem to suffer from limitations in those activities that might exacerbate an asthma attack and make them feel uncomfortable.

A limitation of this study is that it does not take into consideration the fraction of exhaled nitric oxide. This is a new tool for the diagnosis and monitoring of bronchial asthma which allows airways inflammation to be measured. It could be interesting, in future studies, to correlate the PAQLQ to the levels of exhaled nitric oxide.

Another limitation of this study concerns the difficulties of some of the younger children in grasping the concept of "last week"; this might, in fact, cause some biases in the completion of the self-administered version of the PAQLQ.

Moreover, it would have been interesting to compare the HRQL of the caregivers, and to investigate their expectations and priorities concerning asthma, as was recently done by Wu and colleagues [25].

In 2002 a study was conducted by Williams & Williams [26] in order to evaluate the usefulness and feasibility of asthma-specific questionnaires in children during routine clinical practice. They investigated correlations between the PAQLQ score, the score of a caregiver's quality of life questionnaire and the assessment of control given by the clinician in a group of 42 children 7 to 17-years old. No correlations were found between the aforementioned parameters, thus possibly affecting treatment planning with the family. When no ideal single parameter is available to monitor asthma control adequately, the administration of a quality of life instrument is an important and quick-to-administer aid to the clinical activity which can supplement symptom reports, objective measures and clinical assessment of asthma control and severity. The same conclusion was reached by Reichenberg & Broberg in 2003 [27], who found correlations between the PAQLQ, caregiver's quality of life, peak flow rate and the parents' reports of child asthma symptoms in a group of 71 7-to-9-year-olds with asthma. Re-administration of the questionnaire at each follow-up visit could be a useful way to keep the child's HRQL monitored over time.

Recently, the use of an electronic version of the PAQLQ was proposed, and it showed promising results for use in routine asthma care [28].

## Conclusion

Interpretation of HRQL data is still debated. Today, patients are much more closely involved in understanding their condition, identifying their own needs, and working with the clinician in developing a treatment plan [29]. Only three studies, to the best of our knowledge, have used factor analysis to determine whether asthma health status is a homogeneous or heterogeneous construct [30-32]; they all identified that asthma is a composite condition. Two of these papers [31,32] include HRQL evaluation in the analysis, and they identified that HRQL is a distinct component of the asthma health status. Although it is interesting to think of HRQL as a separate facet of this condition, further analysis is necessary to fully understand the role of HRQL assessment in routine practice when dealing with asthmatic children.

## Abbreviations

HRQL: health-related quality of life; PAQLQ: Pediatric Asthma Quality of Life Questionnaire; FVC: forced vital capacity; FEV_1_: forced expiratory volume at the first second; FEF_25–75_: forced expiratory flow between 25% and 75% of vital capacity.

## Competing interests

The authors declare that they have no competing interests.

## Authors' contributions

GR made substantial contributions to the conception and design, acquisition of data, interpretation of data; he also critically revised the manuscript and gave his final approval of the version to be published. AD was substantially involved in the study design, acquisition and interpretation of data, manuscript drafting and revision, and gave her final approval of the version to be published. EB contributed to the study design, data analysis and interpretation, critical revision of the manuscript and gave her final approval of the version to be published. BB was involved in acquisition of data and gave her final approval of the version to be published. AG was involved in data acquisition and gave her final approval of the version to be published. MM contributed to the study design, data interpretation, critical revision of the manuscript and gave his final approval of the version to be published.

## Pre-publication history

The pre-publication history for this paper can be accessed here:


